# Supplementary material for: Feasibility and Preliminary Effects of Acupuncture for Cognitive Dysfunction in Diverse Cancer Survivors: A Pilot, Randomized, Placebo-Controlled Trial
Source: Curr Oncol. 2025 Jan 1;32(1):27. doi: 10.3390/curroncol32010027 (PMC11763785; doi:10.3390/curroncol32010027)
Supplement: Supplementary file 1 [file curroncol-32-00027-s001.zip › curroncol-3346195-supplementary.pdf]

Supplementary Materials

Table S1: Study Protocol Amendments

Amendment Version	Date	Details
Original	5/14/2019	
1	7/10/2019	• Adjusted treatment schedule to 10 sessions over 10 weeks, rather than 8 weeks<br>• Changed primary endpoint from week 8 to week 10<br>• Allowed acupuncturists to add or remove up to 6 points from the core & supplementary points<br>• Clarified sham procedures<br>• Inclusion and exclusion criteria refined<br>• Added Study Allocation Belief Survey to assess blinding success
2	10/30/2019	• Updated exclusion criteria to exclude participants who have received acupuncture for symptom management in the past 3 months instead of 12<br>Study allocation will be based upon prior chemotherapy in the last five years
3	11/27/2019	• Corrected timepoints for outcome measure collection.
4	1/9/2020	• Added randomization letter to unblind patients with after week 16
5	2/17/2020	• Changed sham procedure to non-skin penetrating<br>• Removed potential confounders (e.g. ambient music) to allow for better separation between specific needling effects and non-specific components of the acupuncture process<br>• Updated acupuncture protocol to align with sham; acupuncturists now instructed to use tape to mark the skin close to acupoints before needle insertion<br>• Unblinded PI for safety and study interpretation
6	9/16/2020	• Modified neurocognitive battery and patient-reported outcomes procedures to allow for remote administration and collection
7	11/2/2020	• Removed taping from real acupuncture procedure<br>• Inclusion criteria updated to include prostate cancer survivors
8	2/17/2021	• Removed WLC to focus on refining sham and real acupuncture procedures<br>• Added 10 real acupuncture treatments as compensation for patients randomized to sham group<br>• Eligibility criteria broadened to include stable metastatic disease; completed treatment at least one month prior to enrollment; 10-year diagnosis window eliminated; 8-week window for somnogenic medication change updated to 4-weeks<br>• Increased treatment window from 7 to 14 days to allow more flexibility in scheduling

9	6/3/2021	• Clarified screening question administration and documentation

Table S2: Real Acupuncture Protocol

**Table S2a: Core Points for Cognition**				
**Point Location**	**Acupuncture Points**			
**Head**	**Bai Hui**<br>GV20<br>(unilateral)	**Yin Tang**<br>GV24.5<br>(unilateral)	**An Mian**<br>EX<br>(bilateral)	**Si Shen Cong***<br>Ex<br>(four points)
**Body**	**Shen Men**<br>HT7<br>(bilateral)	**San Yin Jiao^**<br>SP6<br>(bilateral)	**Tai Xi^**<br>KI3<br>(bilateral)	**Zhong Wan**<br>CV12<br>(unilateral)
**Ear**	**Hippocampus**<br>(unilateral)	**Shen Men**<br>(unilateral)		

* If Insomnia Severity Index (ISI) score <8 after Week 4, consider adding these points.^ If ISI score <8 after Week 4, consider 2 Hz electro-stimulation at these points.

**Table S2b: Supplementary Points for Co-Morbid Symptoms**		
**Co-Morbid Symptom**	**Acupuncture Points**	
**Fatigue**	**Zu San Li**<br>ST36<br>(bilateral)	**Qi Hai**<br>CV6<br>(unilateral)
**Psychological Distress**	**Tai Chong**<br>LV3<br>(bilateral)	**Shen Ting**<br>GV24<br>(unilateral)

Table S3: Sham Acupuncture Protocol

**Sham point**	**Point Location**
Upper Arm, UAI	On anterior upper arm, 5 cun proximal to cubital crease, on the bulge of the biceps brachii
Forearm, FI	On the radial styloid process
Hand, H1	On the head of the 3rd metacarpal bone at the dorsal surface
Thigh, TI	On the bulge of the rectus femoris, 5 cun above the middle of the superior border of the patella
Leg, LI	On the anterior crest of the tibia, 7 cun below the base of the patella
Leg, LII	On the medial aspect of the tibial plateau, 3 cun below the base of the patella
Ankle, AI*	On the medial malleolus

* The lateral malleolus is another option if this area is more accessible
